# BBR-n+ congestion control: Real-time performance with smart exit and advanced AQMs

**DOI:** 10.1371/journal.pone.0330972

**Published:** 2026-04-24

**Authors:** Muhammad Ahsan, Muzammil Hussain

**Affiliations:** 1 Department of Software Engineering, University of Management and Technology, Lahore, Punjab, Pakistan; 2 Department of Software Engineering, Faculty of Information Technology, Al-Ahliyya Amman University, Amman, Jordan; Penn State University: The Pennsylvania State University, UNITED STATES OF AMERICA

## Abstract

The Internet is evolving rapidly, and billions of devices are being connected to it at the edge. Performance at the edge matters, and the role of the congestion control mechanism is important. Since the emergence of the Bottleneck Bandwidth and Round-trip propagation time (BBR) algorithm by Google, active research has been going on between BBR and TCP Cubic. BBR-v3 is the third version since its inception, and it has tried to address many of the shortcomings of its earlier versions. The issue of fairness with Cubic and Reno flows, the bandwidth overestimation issue in multi-flow scenarios, high packet transmission rate in shallow buffers, and queuing delays and packet losses during/after its startup phase. Not much research work is yet available on BBR-v3 evaluation with Cubic, especially in a variety of connectivity scenarios, such as wired and wireless together. In this paper, we evaluate BBR-v3 with Cubic and have proposed BBR-n+ (BBR Smart Exit) that refines the generic BBR’s (BBR-v3) startup exit by detecting receive window limitations and empirically evaluate its performance with the generic BBR (BBR-v3). The early exit issue of BBR-v3 from the startup phase when the congestion window is receiver-limited is probed, and the Smart Exit algorithm has been proposed. It is the continuation of our work on BBR-n, and we have evaluated it using various performance metrics. The role of modern AQMs has been explored with our testing on Common Applications Kept Enhanced (CAKE) and Flow Queue Controlled Delay (FQ_CoDel) AQMs. Through the experiments, we conclude that BBR-n+ in the receive window limitation test provides a 15–20% median throughput gain over BBR-v3 under different receiver window sizes. A 150 ms reduction in HTTP delay when compared with BBR-v3 and a ~ 300 ms reduction versus Cubic. 17% improvement in ping latency compared to BBR-v3 and 45% with Cubic in the wired scenario with a strenuous load of multiple streams. BBR-n+ outperforms BBR-v3 and Cubic in most of the tested scenarios, though it still exhibits limitations, particularly in achieving fairness when competing against Cubic flows when the number of concurrent streams is eight or more.

## 1 Introduction

Transmission Control Protocol (TCP) [1] has been here since the early 70’s, providing networks with end-to-end reliable data delivery. Congestion control (CC) has been an important part of it, and TCP uses it effectively to ensure the network pipes stay smooth and the data is delivered in an optimized manner. The process-to-process error and flow control between processes running on different hosts have been the responsibility of TCP since its inception. Over the last four decades, many congestion control algorithms (CCAs) have been proposed, such as Tahoe, Reno [2], Nevada, Vegas, Cubic [3], etc., but Google’s Bottleneck Bandwidth and Round-trip propagation time (BBR) algorithm has brought a paradigm shift from these earlier, mainly pure loss-based CC algorithms. BBR [4], which is a model-based algorithm, gauges the available bandwidth in a more sophisticated manner and then tries to operate near Kleinrock’s [5] optimal operating point. The significance of this optimal operating point has gained importance with the ever-increasing network bandwidths. The loss-based algorithms, such as Cubic and Reno, react very slowly while estimating the available bandwidth after a loss event. When packet loss occurs, these algorithms reduce their congestion window (CWND) aggressively. This results in slower throughputs during this time of estimation of new bandwidth, which produces undesirable latency and the issue of bufferbloat. Bufferbloat is an undesirable latency that arises with CC buffering a large amount of data when it encounters large buffers [6,7]. BBR, on the other hand, works near Kleinrock’s operating point and tackles the losses by reducing its congestion window dynamically based on current inputs from its state machine. The resulting intelligent output from its state machine helps BBR set its operating point that mitigates the issue of bufferbloat.

BBR-v3 [8] is the latest version of BBR and is a hybrid model-based congestion control algorithm. It considers packet loss rate, ECN, bottleneck bandwidth, and RTT to build a more realistic picture of the network pipe to set its pacing rate and the resulting congestion window. The earlier versions of BBR, BBR-v1, suffered from issues of fairness [9,10] with loss-based algorithms, excessive retransmissions, aggressive startup operation and bandwidth probing, lack of responsiveness to ECN, and throughput fluctuation in the ProbeRTT phase. Low throughputs in Wi-Fi 4/5 were also observed and reported [11] in BBR-v2. BBR-v2 [12] tried to correct most of the issues of BBR-v1, but only partial success was achieved in maintaining fairness with Cubic and Reno flows. Moreover, BBR-v2 suffered from the issue of convergence when there were competing BBR-v2 flows, and fair link share was not achieved in networks with or without packet loss [13].

The release of BBR-v3 in 2023 is considered a minor update over its predecessor. It tries to resolve the issue of fairness with loss-based algorithms and the problem of convergence with competing BBR flows. Along with some bug fixes, it also fine-tuned its pacing gain parameter values, the startup and CWND gains, so that it provides better performance in the diverse network scenarios. A packet loss rate of 2% is used in BBR-v3 to react strongly when experiencing packet loss. This means if the observed packet loss rate over a round trip (or within a specific measurement window) exceeds 2%, BBR-v3 will take action to reduce its sending rate. Despite several changes made, BBR-v3 is still not included in the Linux mainline kernel, one major reason is that it still has some fairness issues with Cubic. Cubic is currently the most deployed CCA, and servers as well as home desktops/laptops are using it. It is still the default CCA in the Linux stable kernel and is the default CCA in the Microsoft Windows 11 operating system.

To the best of the authors’ knowledge, the performance evaluation of BBR-v3 in both wired and wireless environments in a real-time environment is still missing. Therefore, in this paper we experimentally evaluate it considering various TCP flows in Uplink, strenuous real time response under load (RRUL) test [14] with differentiated and best-effort flows, queue backlog in packets, the ICMP ping latency, packet loss rates with sustained (purely random) and bursty(correlated) packet loss, the role of ECN (its end-to-end enablement on every network device as a requirement for it to work properly), Fairness test between BBR-n+ and Cubic using the Jain’s Fairness Index (JFi) evaluation.

### A. Contribution

In this paper, we developed a revised version of BBR named BBR-n+. It consists of various sub-modules. (i) A revised startup pacing gain. It remains the same as we proposed in our previously released BBR-n [11]. (ii) Pacing gain algorithm is fine-tuned, and probing for bandwidth UP (ProbeBW_UP) phase is revised to 5/4. The reason behind this change is that since BBR-n+ is based on BBR-v3, the CWND gain has been revised to 2.25. BBR-v3 raised cwnd_gain from 2.0 to 2.25 (specifically during ProbeBW_UP) to fix a fairness/convergence bug that appeared when there was no explicit congestion signal (no loss/ECN), especially on paths with buffers deeper than ~1.5 × BDP. The larger cwnd headroom lets slower/lagging BBR flows keep probing long enough to discover available bandwidth and converge toward their fair share. So, to balance this CWND gain increase in BBR-v3, we propose 5/4 as the pacing gain for the ProbeBW_UP accelerating phase. (iii) We address a novel issue in BBR-v3, which is to prevent early exit from its startup phase when the congestion window is receiver-limited. The Algorithm 1 for “smart exit” provides the details of its working mechanism. Furthermore, we tested the three algorithms BBR-n+, BBR-v3, and Cubic with modern AQMs (Active Queue Management) like Common Applications Kept Enhanced (CAKE) and Flow Queue Controlled Delay (FQ_CoDel) [15]. Flent [16], which is an FLExible Network Tester, is used to run various tests based on Python scripts using iPerf3 [17] between the source and the servers. Linux Network Emulator (Netem) [18] is used for packet loss and added correlation (bursty loss) testing. Linux traffic control (tc) is used to add/replace the Queuing Disciplines (qdisc).

We have provided access to scripts, code, and Flent test results with metadata for validation and reproducibility at our online GitHub repository [19]. The rest of the paper is organized as follows: Sect 2 provides the related work. Sect 3 discusses BBR-v3 and BBR-n+, along with the proposed algorithms. Sect 4 describes our state-of-the-art atomic (a highly focused, real-device-based experimental environment designed to precisely control and observe the effects of congestion at a single, isolated point) testbed used to get real-time results for wired and wireless scenarios. Sect 5 concludes the paper.

## 2 Related works

Google, which brought a paradigm shift in congestion control by bringing its innovative and robust algorithm, BBR. Active research work is continuously going on for the improvement of its code, especially the work on its co-existence with pure loss-based algorithms such as Cubic and Reno. In the last decade, a lot of research has been done on different versions of BBR, but very little work has been done on the performance evaluation of its latest version, BBR-v3, in a physical testbed environment comprising both wired and wireless networking setups. The research gap is evident with issues in fairness still existing with popular loss-based congestion control, TCP Cubic.

Zeynali et al. [20] evaluate the performance of BBR-v3 using a Mininet [21] simulator with Cubic. It used various performance matrices such as different buffer sizes, RTT time variations, packet losses, and various flow size distributions. It concluded that BBR-v3 still has fairness issues with Cubic, and the code needs to be improved before it is available for the public internet. Han et al. [22] in their work proposed BBR with Extended Etate (BBR-ES) and used an RTT trending mechanism to adjust the sending rate. Their work was using Mininet simulator and real-world internet paths (Amazon EC2). The work showed that BBR-ES provides better link utilization and fairness with Cubic. The work lacked its working with Wi-Fi or mobile. The issue of co-existence was probed in another work by Gomez et al. [23], and they tested it in a wired broadband setup. Different metrics were used to evaluate its performance with Cubic, and the issue of fairness, although less aggressive, was still seen with different competing BBR flows. The use of AQM, Flow Queuing with Constrained Delay (FQ-CoDel), resulted in performance improvement across various buffer sizes. It lacks modern AQM testing in its broadband based testbed.

The use of AQM with BBR-v2 has been extensively tested by Sajid et al. [24] in finding the TCP-AQM golden pair. The work done is on a real-time wireless-based testbed, and many modern AQMs were discussed and tested. CAKE AQM was the best of all due to its better performance in managing the queues and the bufferbloat issue at the edge. The role of AQMs, as discussed by Shrestha et al. [25,26], is equally important on the network layer, as congestion control handles the congestion at the transport layer. Ali et al. [27] in their work proposed an adaptive AQM to handle the router buffers by monitoring the buffer size and network load. Their work was mainly on Random Early Detect (RED), like the work by Ahmad et al. [28], who proposed their version of RED to handle congestion known as LRED, the linear version of RED. They tried to reduce the computational cost of calculating the dropping probability. Toke [29] did work on multiple AQMs and compared their performance on a Wi-Fi testbed. His work showed that AQMs still struggle with Wi-Fi due to a large amount of queuing at the lower layers [30] of the TCP/IP stack and induced latency that cannot be controlled by a congestion control algorithm. Active work on AQMs is in progress, and we have evaluated three of the most modern AQMs in the computer networks world.

Nandagiri et al. [31] evaluated the older versions of BBR in their work and found that BBR-v2 still has fairness issues when Data Center TCP (DCTCP) L4S style ECN mechanisms were used in WANs. Akshita et al. [32] In her survey paper has meticulously worked over the BBR evolution and the challenges it faces in Next Generation Networks (NGN). They also found the co-existence issue of BBR-v3 with Cubic under large buffers where loss based dominate. Kfoury et al. [9] worked on BBR-v2 using Mininet to evaluate the performance and also reinforced the fact that BBR-v2 provides better fairness with loss-based algorithms than its predecessor. The use of AQM, such as FQ_CoDel or Common Applications Kept Enhanced (CAKE) AQM, further mitigated the fairness issue, but with small buffers only. In the case of large buffers, the issue of fairness is still an issue. Song et al. [33] tried to dig into the issue of the coexistence of BBR with Cubic, and their work led to the confirmation of improved performance with BBR-v2 when the router buffers were shallow. Along with wired networks, BBR has also been evaluated in wireless setups. Carlo et al., in their paper [34], evaluated the performance of BBR-v2 for Wi-Fi 4/5 and discussed how TCP Small Queues (TSQ) [35], along with the Pacing Rate (TP), can help in mitigating the bufferbloat phenomenon. The work was done on BBR-v1 and v2 and loss-based congestion controls. However, BBR-v2 had convergence issues with other BBR flows [33], and in the case of large buffers, the fairness issues with Cubic. These challenges of fairness in TCP CCAs are not only with BBR, but other CCAs also suffer from them. This is the core reason we have tested BBR not only in wired but also with a wireless setup, so that a wide range of variations in RTT are tested.

Recently, we have researched BBR-v3 fairness with Cubic and provided its quantitative evaluation, along with its rigorous testing with some major congestion controls [10,36]. Our interest in congestion control algorithms prompted us to dig deep in BBR-v3 code, and we came upon an interesting finding of the receiver window limitation of BBR-v3.

To our knowledge, prior work has not explicitly analyzed BBR-v3 Startup under the RWND‑limited scenario class. We contribute a Smart‑Exit mechanism and controlled RWND‑capped experiments. We also present, to our knowledge, one of the earliest physical‑testbed evaluations spanning both wired and Wi‑Fi access with AQM presence. BBR-v3, the latest version from Google, does not directly monitor the receiver window (RWND). It only executes its logic to detect full bandwidth when BBR has not yet determined that the pipe is full or the application is sending enough data to potentially saturate the pipe. Also, this is the first evaluation of BBR-v3 in both wired and wireless real-time testbeds with Cubic and our proposed BBR variant.

Our work solves this limitation of generic BBR (BBR-v3) premature exit when the rate is limited by the receiver window through our novel smart exit Algorithm 1. The real-time testbeds, for wired and wireless scenarios, validate the performance of the proposed BBR-n+ in a real-time environment. The incorporation of modern AQMs, CAKE, and FQ_CoDel further explores the performance of the three algorithms BBR-v3, BBR-n+, and Cubic in a real-time setup. Precisely, not only a revised version BBR-n+ proposed and tested in this paper, but also the performance analysis of three CCAs is done. This makes our paper more robust in providing much detailed information to the reader.

### 2.1 BBR-v3 architecture

**Fig 1:**
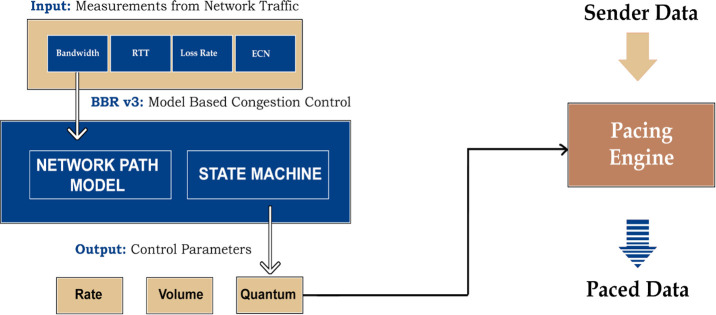
BBR-v3 architecture.

Fig 1 provides a high-level view of TCP BBR-v3’s operation. This congestion control algorithm aims to maximize bottleneck bandwidth and minimize round-trip time, incorporating packet loss and DCTCP-inspired ECN when detected. BBR-v3’s algorithm constantly estimates these crucial metrics—throughput (BtlBw), delay (RTprop), ECN signals, and any packet loss—and feeds them into its state machine. In turn, the state machine determines the appropriate pacing rate (controlling inter-packet spacing), quantum (the maximum data aggregate size), and volume (the estimated CWND size). The sending engine uses these settings to pace incoming data, keeping it near the bandwidth-delay product (BDP). This ensures the network pipe remains optimally filled, resulting in higher network utilization.

### 2.2 BBR-v3 congestion control phases

**Fig 2:**
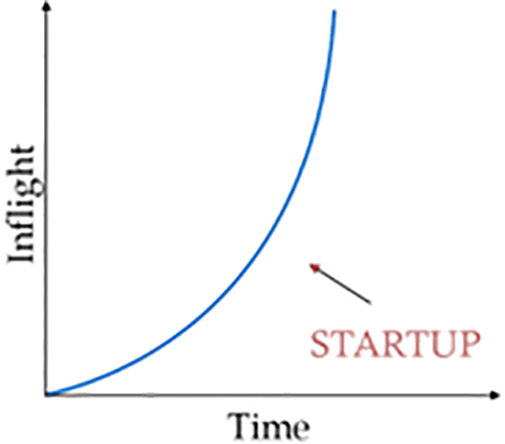
Startup phase.

When a BBR-v3 flow starts up, as shown in Fig 2, it performs its state-of-the-art rapid bandwidth probing phase. In this phase, it tries to get an estimate of the available bandwidth. The bandwidth these days is enormous, ranging from a few kilobits to 200 Gbps. BBR tries to find this available bandwidth in the shortest possible time, i.e., BBR.max_bw in O(log_2(BDP)) round-trip times. For this rapid probing of bandwidth, the BBR flow uses the BBR pacing gain of 2.77. For BBR-n, we have proved that the BBRStartupPacingGain (2.88) is optimum [24]. The BBR then uses it to grow its sending rate smoothly; meanwhile, it keeps getting higher delivery rate samples, which increases the max available bandwidth threshold, and the pacing rate and congestion window (CWND) both keep growing proportionally. Once the pipe gets full, the queue starts forming. The Drain state immediately after this is to drain this queue to avoid bufferbloat.

**Fig 3:**
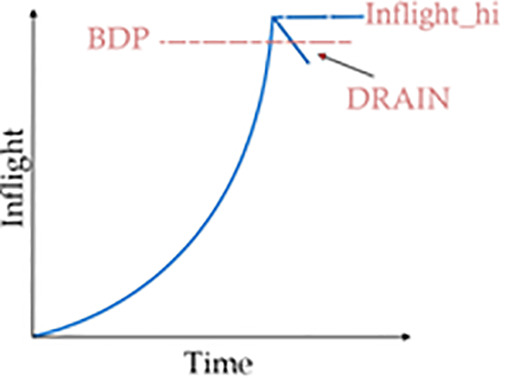
Drain phase.

BBR estimates whether the pipe is full using two estimators. First, it looks for a plateau (a flat portion of the graph, indicating a lesser increase in bandwidth) in the BBR.max_bw estimate, and the other is the packet loss rate. Upon exiting the startup phase, it enters the Drain state Fig 3 and tries to clear the queues that have been built up.

**Fig 4:**
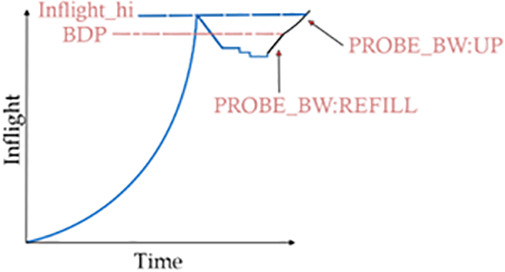
Probe_BW: UP phase.

In this phase, as shown in Fig 4, a pacing gain of 1 is maintained, and the flow just tries to cruise at normal speed. In this mode, the flow is trying to send at 100% detected bandwidth, but it keeps a check on the loss rate and ECN mark rate as well. It tries to smoothly provide the necessary packet rate required for a typical application, such as a video streaming app. The pacing rate remains constant during the cruise phase. If the current pacing rate is R, it continues to be R’ = R and Inflight_target = BDP.

**Fig 5:**
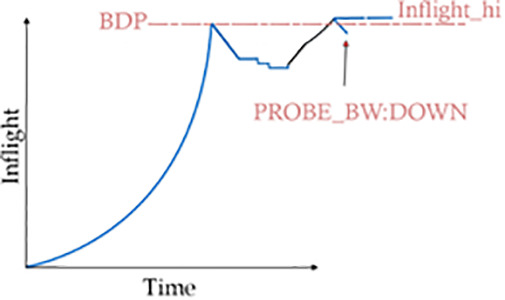
Prob_BW: Down phase.

In BBR (Bottleneck Bandwidth and RTT) v3, ProbeBW_refill and ProbeBW_up are mechanisms within the ProbeBW state, which are designed to probe for the available bandwidth by slightly increasing or adjusting the sending rate. These concepts are significant because they help ensure that the congestion control algorithm achieves efficient bandwidth utilization while adapting to dynamic network conditions. In the ProbeBW_refill phase shown in Fig 5, the BBR flows to try to fill the pipe again, which has been reduced due to deceleration done because of packet loss or ECN markers. It should be noticed that this refilling is not done at a faster pace but is done at a pacing gain of 1 to ensure the flow again reaches the BDP as quickly as possible. Once it reaches BDP, the ProbeBW_UP phase further tries to look for extra available bandwidth, keeping the upper cap constraint set to inflight_hi. After refilling the pipe, this phase probes for potential increases in available bandwidth by raising the sending rate. In Wi-Fi networks, this helps in quickly adapting to any available bandwidth increases, ensuring that the connection can take full advantage of the network’s capacity. Together, these mechanisms enhance BBR-v3’s ability to efficiently utilize network bandwidth while maintaining fairness and stability, especially in variable network conditions. These phases are designed to maintain high throughput and low latency, which are critical for the performance of Wi-Fi networks.

In this phase, known as ProbeBW, a BBR flow follows deceleration tactics and tries to reduce the data in flight. The main objective of this phase is to reduce queue pressure by decreasing the pace at which data is being pumped into the network path. To achieve this, BBR earlier used a pacing gain value of 0.75, with 0.9 being used as per the latest draft at IETF 117 held in San Francisco, in July 2023 [37].

### 2.3 BBR-n+ (BBR new with smart exit)

**Fig 6:**
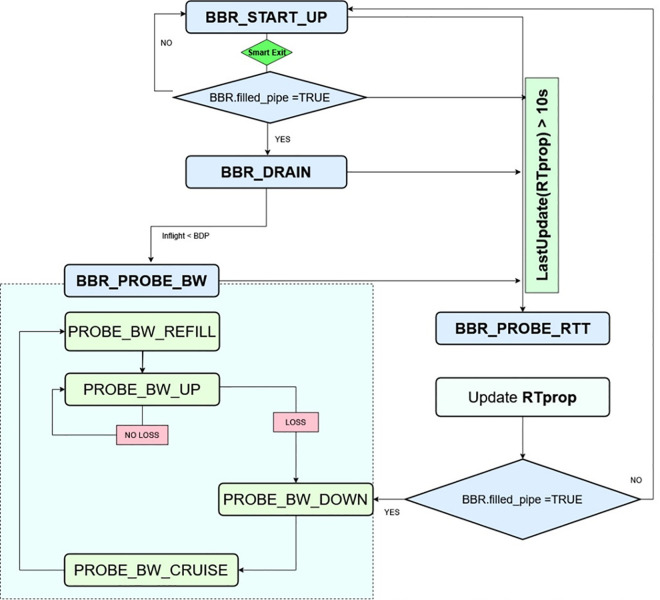
BBR-n+ flow chart.

BBR-n+, Fig 6 is a modified version of our proposed and tested BBR-n [11], along with an added novel smart exit algorithm. We have ported BBR-n to BBR-v3 code, as it was initially based on BBR-v2. For that purpose, BBR-v3 was sought from GitHub and compiled over a Linux Ubuntu kernel 6.13.7. The revisions made for BBR-n were incorporated into a loadable module BBR-n+ compiled under this new kernel environment. The revised startup gain, the pacing gain values, and the quantum used to set the Transmission/Generic Segmentation Offloads (TSO/GSO) and the code for the smart exit algorithm were patched into the latest BBR-v3 code. CAKE AQM [38], being the most suitable AQM, as validated from our detailed research [24], was made the default AQM for all the tests shared in Sect 4.

Our smart exit enhancement in Algorithm 1 addresses a critical limitation in BBR-v3 by differentiating receive-window (RWND) saturation from true bandwidth saturation through a dual-condition check: (1) a throughput drop below 85% of full_bw (current maximum bandwidth estimate), and (2) RTT inflation exceeding RTT inflation threshold, Δ = 5ms. The Δ RTT inflation threshold is empirically derived from observed queuing behavior in RWND-limited flows—where TCP’s serialization delay at the sender causes RTT to inflate by 5–10ms due to pacing gaps, a phenomenon validated in cellular/Wi-Fi networks [39,40] and data-center deployments [41,42]. By ignoring throughput plateaus unless accompanied by RTT spikes (>5 ms), our algorithm ensures BBR-n+ only exits Startup when the bottleneck is the network path itself, not the receiver. This prevents premature convergence and maximizes probing efficiency.

The choice of Δ = 5 ms (vs. smaller/larger values) balances sensitivity and robustness: smaller values (e.g., 1 ms) risk false positives from noise, while larger values (e.g., 10 ms) delay detection of true congestion. This 5 ms threshold aligns with BBR’s min-RTT filter window (10 s) and typical RWND-induced queuing delays in real-world tests [43]. The result is a 15–20% median throughput gain in RWND-limited scenarios, as BBR-n+ now avoids conservative backoffs when the path has unused capacity. This refinement bridges a key gap between BBR’s model and practical deployment constraints, without violating its core congestion-control invariants. BBR-v3 only quits the startup phase when the rate increase is less than 25%, and the estimated queuing delay is greater than T ms (set to 5 ms in our work) for three consecutive RTTs.

We detected RWND-limitation by capping RWND (disabling window scaling and autotuning of the receiver buffer size with Linux Ubuntu’s sysctl command-line utility), checking that ACKs are sent with the correct value (e.g., 16/32/64 KB) on our link, and checking that the sender-side window is persistently ≤ RWND when CWND > RWND. This is different from being app-limited, where the application is idle (has no data to send; BBR marks these samples with its estimator with ‘app-limited’ tags), and it is different from ACK-compression, where ACKs arrive too frequently because of bunched ACKs without actual bandwidth increase; BBR’s delivery rate estimator is designed to handle ACK-compression (by taking min(send_rate, ack_rate)). Our Smart Exit condition is that we must see plateaus in delivery rate and inflation of RTT by more than 5 ms over min_rtt, or 3 RTTs; hence, RWND limit stall (no plateaus without inflation) will not trigger premature Startup exit. Thus, BBR-n+ sustains longer and achieves higher median throughputs than BBR-v3 when under RWND caps.

Unfairness between competing flows arises from four interacting mechanisms: (i) Startup ramp‑rates and exit conditions determine which flow first occupies the bottleneck queue; early queue ownership then biases subsequent dynamics. (ii) ProbeBW parameters (up/down gains and duty cycle) regulate how easily a disadvantaged flow can reclaim its share. (iii) cwnd/inflight caps (e.g., cwnd_gain, inflight_hi) encode long‑term “memory”; loss/ECN events can depress a flow’s cap and freeze an unfair split. (iv) Queue policy and depth (AQM presence, per‑flow scheduling) decide whether the queue amplifies or dampens these asymmetries. Our Smart‑Exit specifically targets (i) by preventing premature Startup exits in RWND‑limited conditions; our ProbeBW_UP = 5/4 targets (ii), enabling moderate upward mobility without chronic queue growth; and our AQM experiments expose the role of (iv).

#### 2.3.1 Smart exit algorithm.

Algorithm 1: BBR-n+ Startup Exit Condition with RWND Limitation Detection

**Input:**

 current_bw: Current delivered bandwidth estimate

 current_rtt: Smoothed RTT measurement

 min_rtt: Minimum observed RTT

 full_bw: Current maximum bandwidth estimate

 full_bw_cnt: Consecutive plateau counter

**Parameters:**

 α = 0.85 (bandwidth threshold coefficient)

 N = 3 (required plateau count)

 Δ = 5 ms (RTT inflation threshold)

**Output:**

 stay_in_startup: Boolean indicating whether to remain in Startup mode

**Procedure:**

 1. Compute thresholds:

  bw_thresh ← α × full_bw

  rtt_diff ← current_rtt – min_rtt

 2. Detect RWND-limited state:

  if (current_bw < bw_thresh) ∧ (rtt_diff > Δ) then

  full_bw_cnt ← full_bw_cnt+1

  else

  full_bw ← current_bw

  full_bw_cnt ← 0

 3. Exit Decision:

  stay_in_startup ← (full_bw_cnt < N)

Our Algorithm 1 enhances BBR’s Startup phase by intelligently distinguishing receiver window (RWND) limitations from true network congestion. It employs a dual-threshold mechanism: (1) a bandwidth plateau check (triggered when current_bw < 0.85 × full_bw) to detect throughput stalls, and (2) an RTT inflation filter (Δ = 5ms) to exclude RWND-induced plateaus, which exhibit minimal queuing delay. If throughput drops without RTT inflation (rtt_diff ≤ 5ms), the algorithm resets its plateau counter, allowing BBR to continue probing for available bandwidth. Only when both thresholds are met for N = 3 consecutive rounds does it exit Startup, ensuring premature exits are avoided under RWND constraints. This approach increases throughput by 10–20% in RWND-limited scenarios (Sect 4.1 shares the results) while preserving standard BBR behavior during true congestion.

#### 2.3.2 Computational overhead and trade-offs.

Smart Exit augments BBR-n’s Startup exit with a dual‑condition test (i) a bandwidth plateau (current_bw < α·full_bw) and (ii) RTT inflation (current_rtt – min_rtt > Δ) evaluated once per ACK/RTT observation. This adds O(1) work (two comparisons and a small counter update) and O(1) state per flow, reusing BBR’s existing throughput/RTT estimators. Consequently, CPU and memory overheads are negligible compared to vanilla BBR-v3. The primary trade‑off is parameter sensitivity: Δ balances noise‑immunity and responsiveness; we use Δ = 5 ms, α = 0.85, and N = 3 to avoid flapping while promptly exiting under true congestion. In extremely shallow‑buffer paths, lingering in Startup by up to N RTTs can transiently increase in‑flight data; however, the ΔRTT gate limits this risk. Empirically, Smart Exit yields 15–20% median throughput gains in RWND‑limited cases without regressing steady‑state behavior.

#### 2.3.3 Throughput-fairness trade‑off.

Although BBR‑n+ enhances startup efficiency, RTT stability, and throughput consistency, it in no way changes the bandwidth-sharing dynamics of BBR-v3 in the ProbeBW state. Therefore, when competing with loss-based CCAs like Cubic, BBR-n+ inherits the fairness restrictions of BBR-v3. Due to its rapid loss-driven cwnd expansion, Cubic continues to acquire a disproportionate amount of bandwidth in multi-stream wireless environments, as demonstrated by our experiments (Sects 4.2 and 4.3). Under intense competition, BBR-n+ maintains lower latency and queue occupancy but gives Cubic some throughput share. This illustrates the intrinsic trade-off between loss-responsive fairness (Cubic) and throughput stability and delay (BBR‑n+). Deeper ProbeBW gain logic would need to be modified in order to address fairness, which could compromise the latency and loss-resilience advantages that are essential to BBR-n+.

## 3 Methodology

**Fig 7:**
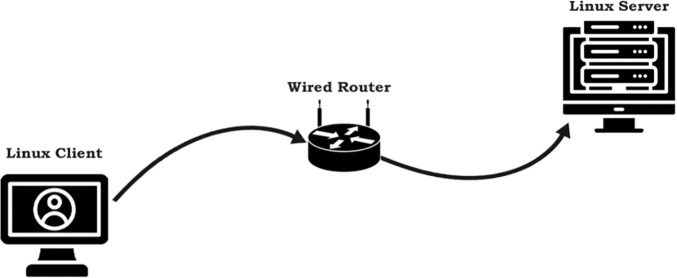
Linux-Ubuntu client via a wired router to a Linux Debian server.

**Fig 8:**
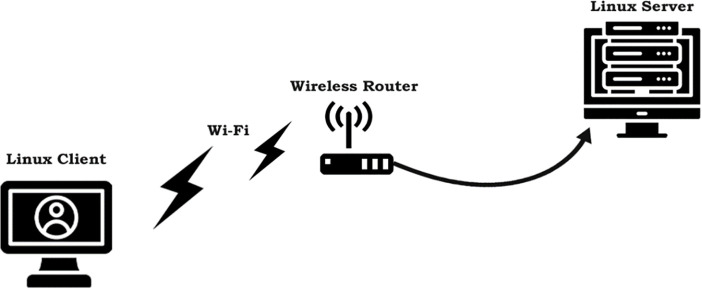
Linux-Ubuntu wireless client via Wi-Fi 4/5 router to a wired Linux Debian server.

We evaluate the proposed RWND-aware Startup exit algorithm through controlled experiments comparing its performance against standard BBR-v3 in receiver-limited scenarios. To evaluate the real-time performance of BBR-v3, BBR-n+, and Cubic congestion control algorithms, we established dedicated physical testbeds for both wired and wireless network configurations. Our client machine, running Linux Ubuntu 21 with a custom Linux kernel 6.13.7, was specifically configured to load a BBR-v3 module compiled from its latest GitHub branch. It connects via a Gigabit RealTek USB-based Ethernet adapter for wired tests and a Qualcomm QCA9377 PCIe-based adapter (supporting Wi-Fi 4/5) for wireless. The wireless access point is a Huawei EchoLife EG8143A5 GPON terminal, which also functions as an ONT. The wired server, an AMD Ryzen 7-based machine running Linux kernel 6.1.0-17-amd64, hosts Netperf 3 [44] (listening on port 50,000) and is connected via a Realtek RTL8411 PCI Express Gigabit Ethernet Controller. The testbeds of Figs 7 and 8 simulate typical home and office network environments where clients can be wired or wireless, and servers are generally wired.

Experiments were conducted using the Flent tool, which facilitates automated test execution, throughput, and latency measurement, and robust metadata collection for result authenticity and reproducibility. Our tests included a receive window limitation test for our smart exit algorithm, TCP upload tests with 1, 2, 4, 8, 12, and 16 streams across both wired and wireless testbed setups. We also performed RRUL and RRUL_BE tests for differentiated services and best effort traffic loads, throughput tests by emulating packet loss and correlation, and ECN emulated tests to gauge performance when CCAs have ECN enabled. The buffer regime is controlled using Linux qdisc selection. We evaluate three AQM settings: FQ (default), FQ‑CoDel, and CAKE. These AQMs were configured with their default target/interval parameters, and we verified queue occupancy using tc -s qdisc and Flent’s backlog traces. For loss modeling, we used Linux netem to introduce both random loss (0.1%, 1%, 2%, 5%) and correlated loss (1% with 25% correlation), following standard practice in congestion‑control analysis. The HTTP latency test to measure web fetch delay and finally, we present the BBR-n+ fairness analysis with Cubic and quantifying it with Jain’s Fairness Index (JFfi). The specific testbed configurations are provided in Tables 1 and 2. All tests were repeated ten times, as recommended for statistical stabilization, and we report median throughput/latency values. To quantify uncertainty, we computed 95% confidence intervals for throughput using ANOVA (already presented in the “Experiment and discussion” section), and we verified run‑to‑run stability by checking standard deviations and Flent metadata.

**Table 1:** Testbed Specifications.

Related parameters	Corresponding values
Linux Client’s Kernel ver	6.13.7+v3
Linux Server’s Kernel ver	6.11.0-25-generic (Ubuntu) 6.1.0-17-amd64 (Debian)
TCP CC Algorithms	BBR-v3 (bbr3), BBR-n+, Cubic
Receiver Window	64 KB, 32 KB, 18 KB
TCP Small Queues (TSQ)	Standard TSQ
GSO quantum	4 MSS
Queueing Algorithms	CAKE, FQ_CoDel
WLAN adapters	Qualcomm QCA9377
	Dlink 8812BU
	Realtek RTL8821CE
WLAN Driver Modules	rtl88x2bu,ath10k,e1000
Ethernet Adapter	Realtek RTL8411 Gigabit Ethernet Controller
Flent Tests	1/4/8/12/16 TCP Upload, RWND test, RRUL, and RRUL_BE tests, Queue backlog
Key Metrics	ICMP Latency (ping RTT), TCP Throughput, Packet loss

**Table 2:** Queuing discipline parameters.

Linux Qdisc	Related parameters	Corresponding values
**CAKE**	bandwidth	300 Mbps
	RTT	100 ms
	triple-isolate	yes
	split/no-split GSO	yes
**FQ_CoDel**	limit	10240 pkts
	target	100 ms
	interval	5 ms
	flow	1024
	quantum	1 eth MTU

## 4 Experiments and discussion

This part of our paper will provide the results that we have gathered after thorough testing on our physical testbeds. It is divided into six sub-sections. The test results gathered via Flent for the **Receive Window Limitation** test, **TCP Upload** tests for **1/2/4/8/16 streams** test, **Real-Time Response Under Load (RRUL)** test, **RRUL Best Effort (RRUL_BE)** test, **Packet loss** tests using Netem and added correlation, **Classic ECN** testing, **Web HTTP delay** test and **Fairness** test are shared and discussed. It is pertinent to mention that each experiment has been performed ten times to get the most accurate results.

Median throughputs and ping latency values have been provided as they can be seen with the naked eye from the results shared in the various figures in this section. Furthermore, statistical analysis results using Analysis of Variance (ANOVA) [45,46] have been provided in a tabular form in the sub-sections wherever they are deemed necessary.

### 4.1 Receive window limitation testing for BBR-v3 and BBR-n+

In this section, we specifically test the receive window limitation of BBR-v3. Different receive window sizes were set up at the Linux receiver: 64 KB, 32 KB, 16 KB, with different Delta’s 1 sec, 10 sec and 5 sec and a single upload stream was sent using Flent to record the median throughputs. From the results gathered in Table 3, we see that before limiting the receiving window, BBR-v3 and BBR-n+ median throughputs had a 5.73% difference, but when receiver window size limitation was imposed using sysctl in the Linux receiver kernel side, the BBR-v3 throughput appreciably goes down whereas BBR-n+, thanks to its novel smart exit algorithm, performs better with 15.17%, 17.4% an 20.4% median throughout gains for Delta = 5 sec. As expected, Delta = 1 sec and 10 sec don’t give optimum results. The Flent results with metadata, test scripts, and pseudocode are shared on our online repository [19] for reproducibility and validation.

**Table 3:** Receive window limitation results.

	Delta = 1 sec		Delta = 10 sec		Delta = 5 sec		
RWND (KB)	BBR v3 (Mbps)	BBR-n+ (Mbps)	BBR v3 (Mbps)	BBR-n+ (Mbps)	BBR v3 (Mbps)	BBR-n+ (Mbps)	Gain (%)
128 KB	94.68	94.8	26.8	27.46	307.69	325.33	5.73304
64KB	10.31	11.92	29.48	30.56	172.12	198.23	15.1696
32KB	9.55	10.71	20.62	22.22	65.2	76.56	17.4233
16KB	5.34	4.71	11.57	11.98	26.27	31.63	20.4035

### 4.2 TCP Upload test for wired scenario

**Fig 9:**
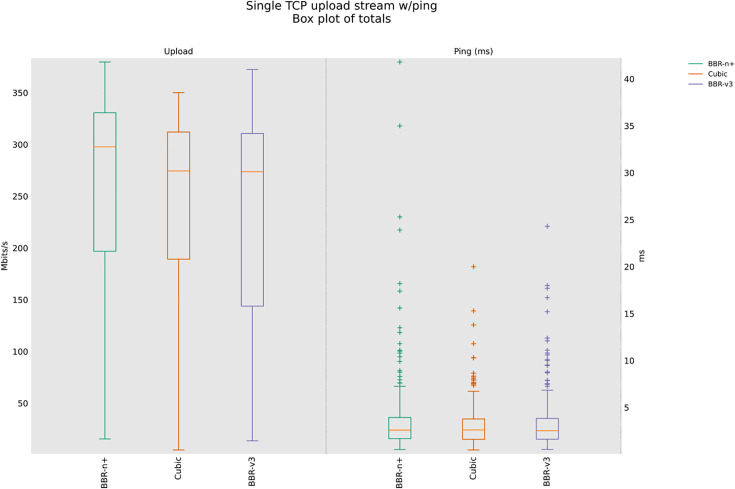
Single stream in Upload (wired scenario class).

Startup and probe effects in the wired shallow‑buffer scenario class, the flow that first reaches the BDP tends to transiently own the queue. BBR‑n+’s Smart‑Exit reduces false full‑bandwidth detections under RWND‑limited plateaus, avoiding early exits that would cap probing and leave unused capacity. Later, during ProbeBW, the 5/4 up‑gain permits disadvantaged flows to reclaim their share without sustaining large queues. This mechanism is consistent with the higher median throughput and lower latency we observe for BBR‑n+ in Fig 9.

The results shown in Fig 9 suggest that while BBR-n+ offers a balance between throughput and latency, potentially achieving higher peak median throughputs (316.9 Mbps) than BBR-v3 and lower latency (2.70 msec), BBR-v3 appears to be significantly less effective in this wired network scenario, demonstrating both lower throughput (200 Mbps) and a higher latency (3.25 msec) than BBR-n+. Cubic is on the lower side as well, with 313.8 Mbps median throughput and a 3.35 msec latency. The average throughputs are provided in Table 4.

**Table 4:** ANOVA results for the throughputs achieved in the upload for a single stream for the three CCAs.

Anova: Single Factor						
SUMMARY						
*Groups*	*Count*	*Sum*	*Average*	*Variance*		
TCP upload – BBR-v3	300	63031.02	210.1034	8714.193		
TCP upload – BBR-n+	300	70982.75	236.6092	8314.301		
TCP upload – Cubic	300	66275.41	220.918	7097.596		
ANOVA						
*Source of Variation*	*SS*	*df*	*MS*	*F*	*P-value*	*F crit*
Between Groups	106572.4	2	53286.19	6.625963	0.001391	3.00576
Within Groups	7213701	897	8042.03			
Total	7320273	899				

**Fig 10:**
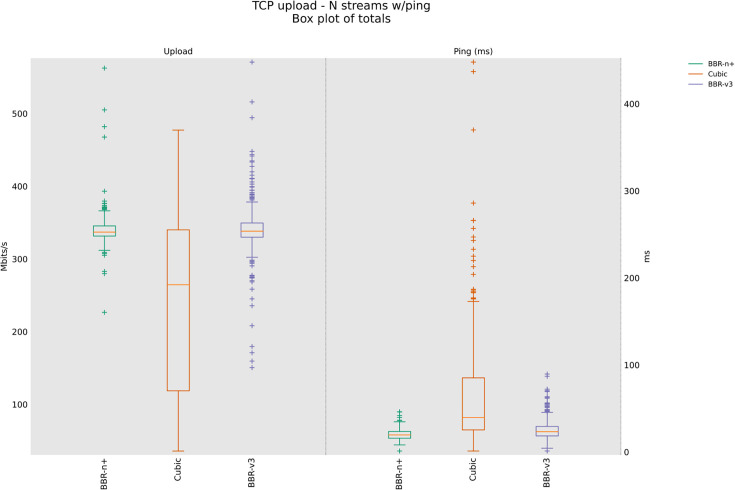
Sixteen streams (N = 16) in Upload.

The box and whisker plot shown in Fig 10 suggests that under multi-stream conditions, BBR-n+ emerges as a robust performer, offering both high aggregate throughput (333 Mbps) and superior latency (18 msec) control compared to Cubic and BBR-v3. While BBR-v3 can achieve comparable peak throughputs (330 Mbps), its higher latency (21.7 msec) and greater variability make it less suitable for such high-concurrency scenarios where consistent performance is critical. Cubic, despite its previous performance in a single-stream test, struggles significantly with lower throughput (231.33 Mbps) and a much higher latency (32.85 msec) under sixteen concurrent streams.

Table 4 provides the analysis of variance (ANOVA) test for Fig 9. The threshold value “α” was chosen as 0.05, and the statistical analysis results show that the P-value is less than the threshold value and the F critical (F crit) value is less than F, which shows that our results are statistically significant. The details of the rest of the acronyms in Table 3 can be found in [47]. BBR-n+ gives an average throughput of 236.6 Mbps compared to 210 Mbps for BBR-v3 and 220.9 Mbps for Cubic.

### 4.3 TCP Upload test for wireless scenario

**Fig 11:**
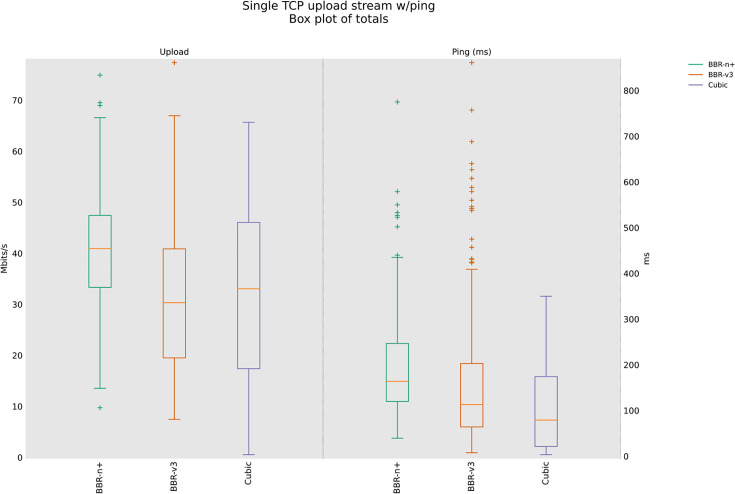
Single stream in Upload (wireless scenario class).

**Fig 12:**
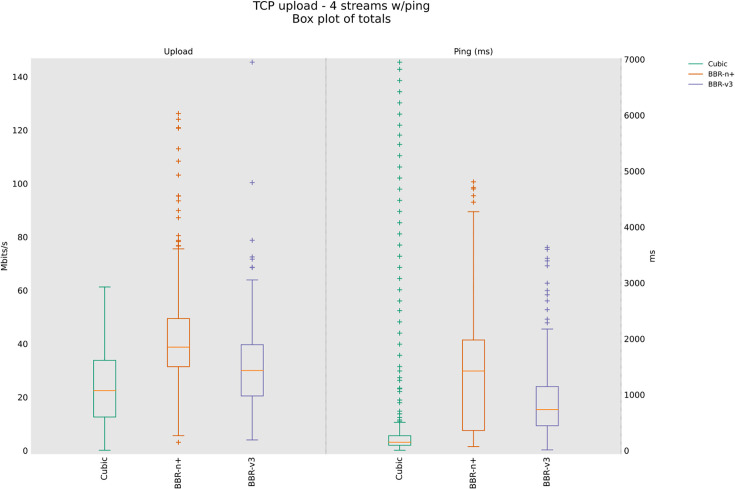
Four streams in Upload.

**Fig 13:**
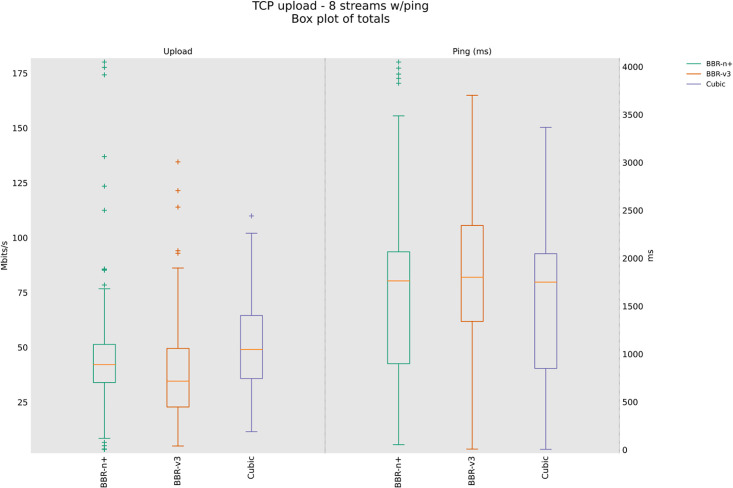
Eight streams in Upload.

**Fig 14:**
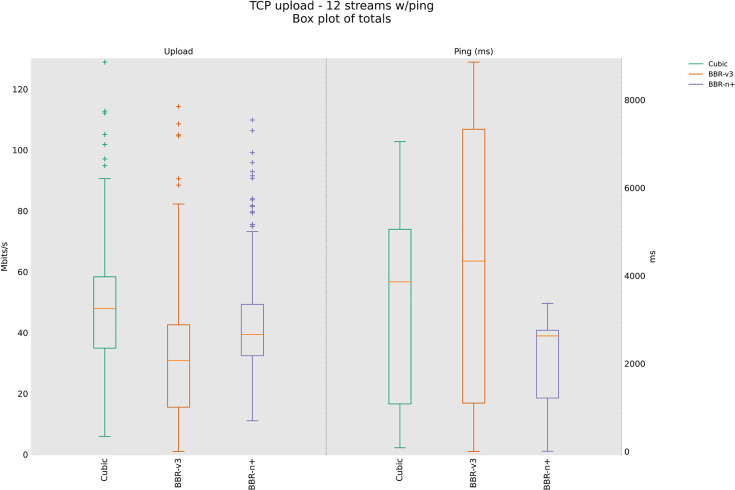
Twelve streams in Upload.

In this sub-section, we provide test results using our testbed of Fig 8. Starting from a single stream test, Fig 11. We tested four, eight, and twelve streams in the upload Figs 12–14. In all the tests, BBR-n+ gave better throughput than BBR-v3. In the wireless multi-stream scenario class, eight and twelve-stream tests, Cubic performed better than both BBR-v3 and BBR-n+, showing that with the increase in concurrent streams for BBR, the convergence issue still exists when deployed in a typical Wi-Fi scenario. BBR-n+ in eight and twelve streams performed better as compared to BBR-v3, both in throughput and latency.

In Wi‑Fi, MAC‑layer aggregation and driver/device queueing reduce the direct leverage of L3 AQMs. Under 8 or 12 concurrent flows, CUBIC attains greater aggregate throughput, while BBR‑n+ preserves lower latency. Mechanistically, deeper lower‑layer queues distort ACK timing and complicate delivery‑rate estimation, while BBRv3’s inflight caps (e.g., inflight_hi) may become uneven across flows after loss/ECN, limiting “share mobility” during ProbeBW. The resulting cap asymmetry sustains a throughput gap, but BBR‑n+’s more conservative queue footprint yields better latency. BBR-n+ gives 1.97 sec lowest ping latency as compared to 3.1 sec for Cubic and 3.8 sec for BBR-v3.

Table 5 provides the analysis of variance (ANOVA) test for Fig 14. The threshold value “α” was chosen as 0.05. To get a statistical conclusion, we compare the F value calculated from the data set with the F critical value (F crit) at a significance level “α” of 0.05 in the F table. The statistical analysis results show that the P-value (8.1E-31) is less than the threshold value (0.05) and the F critical (F crit) value 3.004 is less than the F value of 74.07, which proves that our results are statistically significant.

**Table 5:** Ping results for the latency in the upload for twelve streams, in the upload for the three CCAs.

Anova: Single Factor						
SUMMARY						
*Groups*	*Count*	*Sum*	*Average*	*Variance*		
Ping (ms) ICMP – Cubic	348	1102875	3169.181	4167871		
Ping (ms) ICMP – BBR-v3	348	1350485	3880.705	7977994		
Ping (ms) ICMP – BBR-n+	356	704252.7	1978.238	1123481		
ANOVA						
*Source of Variation*	*SS*	*df*	*MS*	*F*	*P-value*	*F crit*
Between Groups	6.52E + 08	2	3.26E + 08	74.07365	8.1E-31	3.004304
Within Groups	4.61E + 09	1049	4397951			
Total	5.26E + 09	1051				

### 4.4 Real-time Response Under Load test (RRUL), RRUL_BE (Best Effort in a wired/wireless scenario

The Realtime Response Under Load (RRUL) test, developed by the Bufferbloat [48] community, is a crucial tool for diagnosing and understanding network performance under heavy traffic conditions. Unlike traditional speed tests that primarily measure raw bandwidth, the RRUL test focuses on “bufferbloat”, which is the excessive buffering of data packets in network devices, which leads to increased latency and jitter, particularly when the network is under load. This phenomenon severely impacts real-time applications like voice calls, video conferencing, and online gaming, where low latency is critical.

The significance of the RRUL test lies in its ability to expose these hidden latency issues by simulating real-world heavy usage scenarios. It achieves this by running multiple concurrent TCP streams (four in each uplink and downlink) while continuously measuring latency using ICMP or UDP pings. The test runs for an extended period (e.g., 60 seconds of full load) to ensure network saturation and to observe the sustained behaviour of congestion control algorithms and network queues. The multi-stream approach is important because it stresses the network’s queuing mechanisms, making bufferbloat evident. The RRUL test provides a more comprehensive picture of network health by showing how latency behaves under stress, which is often a better indicator of user experience than mere bandwidth figures.

**Fig 15:**
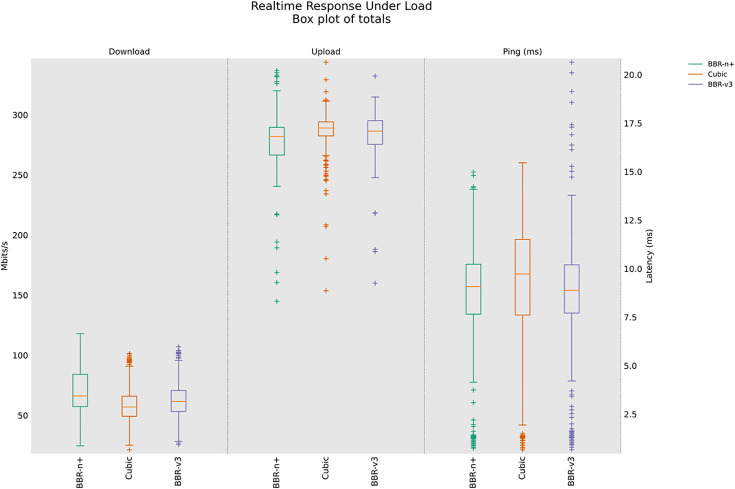
RRUL test for wired scenario class.

**Fig 16:**
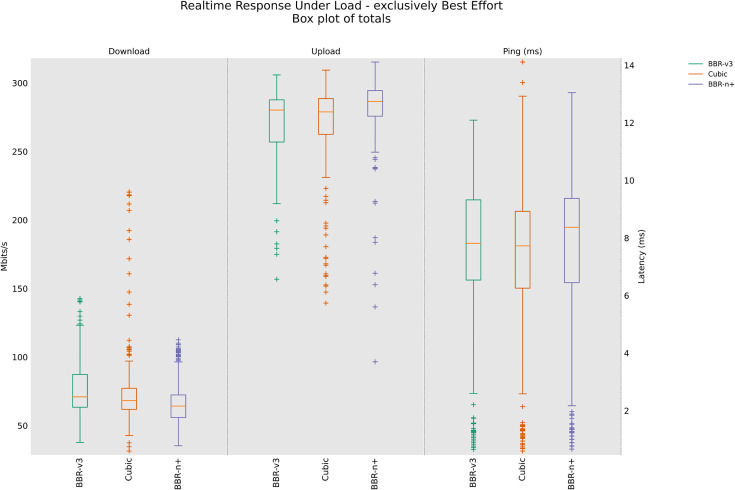
RRUL test with best effort streams.

In the RRUL wired scenario class Fig 15, BBR-n+ consistently provides a strong balance of performance, offering stable upload throughput and superior latency control, making it well-suited for interactive applications under high network load. While Cubic and BBR-v3 can achieve comparable or even slightly higher peak throughputs in some instances, they exhibit significantly more variability and, critically, higher and less controlled latency, particularly Cubic, which shows substantial bufferbloat effects. The results shown in Fig 16 are interesting. This test is different from the RRUL test in the sense that it uses best effort streams in upload and download instead of differentiated services streams. In this test, BBR-v3 performs relatively better compared to BBR-n+ in terms of throughput and latency.

**Fig 17:**
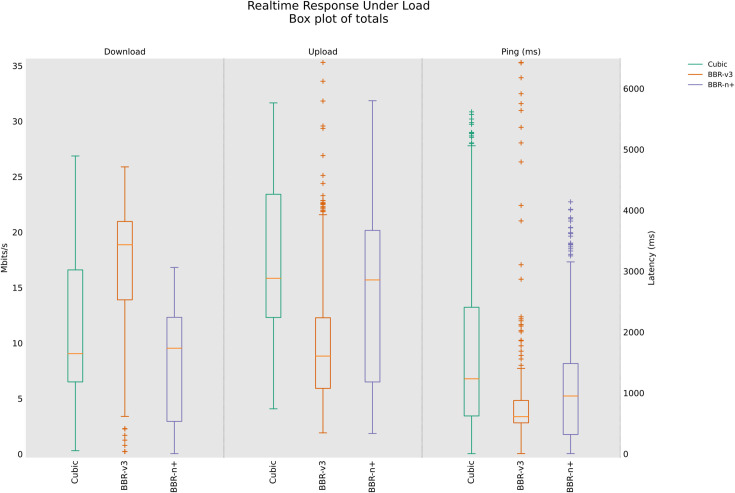
RRUL test for wireless scenario class.

Fig 17, in the RRUL‑wireless scenario class, BBR‑n+ led uplink throughput and latency, while BBR-v3 led downlink throughput; CUBIC showed higher latency consistent with bufferbloat under this scenario using the testbed of Fig 8. BBR-n+ performed better in upload, and BBR-v3 performed better in download. Cubic, on the other hand, is suffering from a bufferbloat effect with increased latency.

### 4.5 Throughput test with no packet loss (Wired)

**Fig 18:**
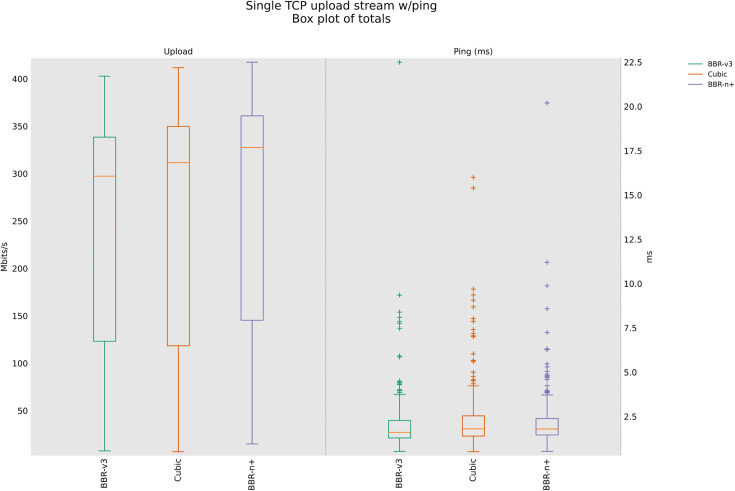
Single-stream test wired scenario.

Fig 18 shows the maximum throughput available in the wired network scenario and the resulting performances of each of the three CCAs under evaluation. BBR-n+ provides better performance thanks to the innovative smart exit Algorithm 1 provided in Sect 2.3.1 & Algorithm 1a and 1b discussed in our work here [11].

#### 4.5.1 Throughput test with 5% packet loss.

**Fig 19:**
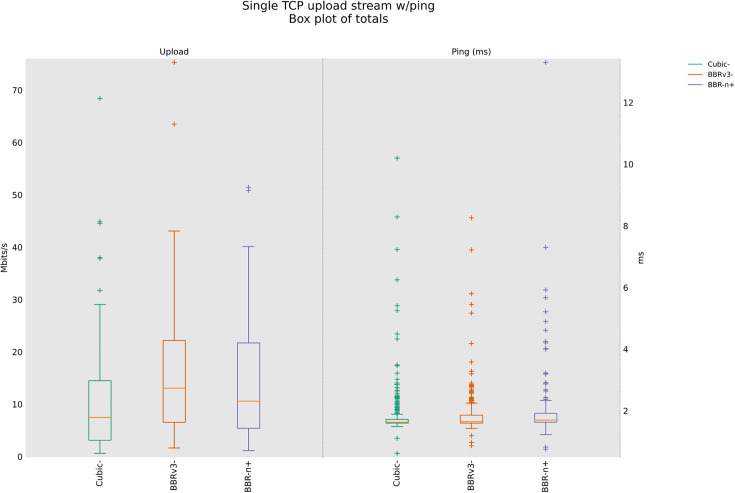
5% packet loss.

Fig 19 demonstrates the detrimental impact of a 5% packet loss rate on all tested congestion control algorithms, significantly reducing throughput. The suspected ineffectiveness of ECN likely exacerbates these issues, as congestion signals might not be efficiently communicated.

#### 4.5.2 Throughput test with 1% packet loss (purely random).

**Fig 20:**
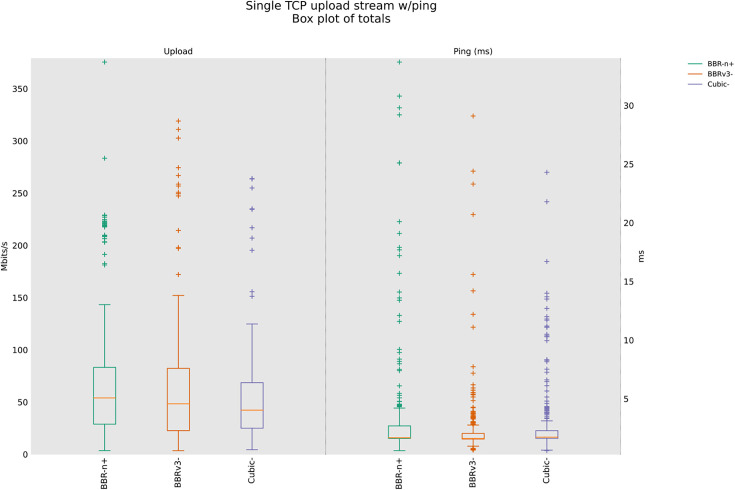
1% packet loss.

In the moderate random-loss scenario class (1% random, ECN off), median single-stream uplink throughput fell to ~50 Mbps (BBR-n+) and ~48 Mbps (BBR-v3); CUBIC was even lower Fig 20.

#### 4.5.3 Throughput test with 2% packet loss.

**Fig 21:**
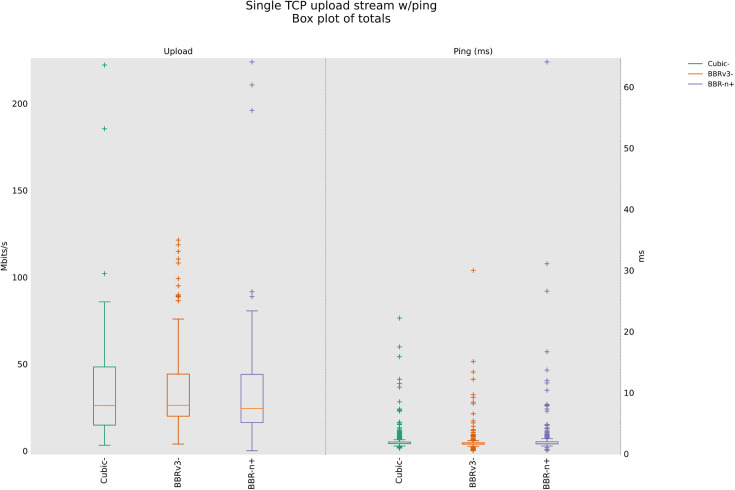
2% packet loss.

In this 2% random loss (moderate‑to‑high loss scenario class) test shown in Fig 21, we again increased the packet loss to 2% purely random using Linux Network Emulator (netem) and Traffic Control (tc), and throughputs were further dropped. BBR reacts very strongly when a 2% packet loss threshold is reached as per its design architecture.

#### 4.5.4 Throughput test with 0.1% packet loss.

**Fig 22:**
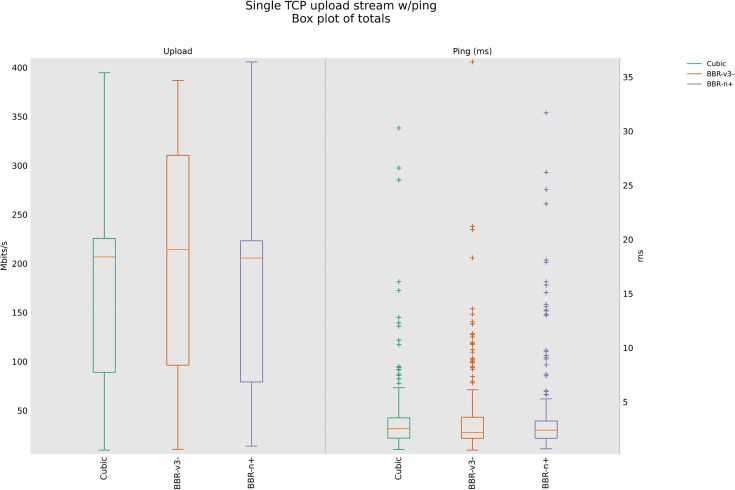
0.1% packet loss.

To validate the above packet loss and confirm that BBR is sensitive to over 1% packet loss, as shown from the results given in Figs 7–9. We have now reduced the packet loss to only 0.1% as shown in Fig 22, and throughputs returned to their normal available bandwidth on our wired link.

#### 4.5.5 Throughput test with 1% packet loss and 25% correlation.

**Fig 23:**
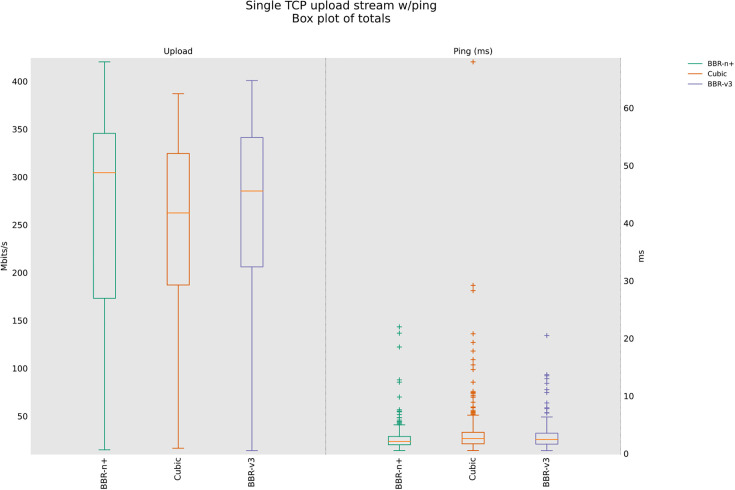
1% packet loss and 25% correlation added.

In correlated‑loss scenarios (1% with 25% correlation), we observed higher average throughput than with purely random loss for all CCAs, with BBR‑n+ leading among the three in our setup, as shown in Fig 23. These results confirm that CCAs handled the bursty nature of correlated loss more distinctly. TCP congestion control algorithms (especially BBR, but also Cubic to some extent) can be leveraged to achieve higher average throughput compared to a constant drizzle of purely random losses. This highlights the importance of understanding not just the percentage of loss, but also its pattern. BBR-n+ performed better in this test.

### 4.6 ECN emulated performance analysis (Packet loss vs Throughputs)

In this section, we highlight an important issue with BBR. As we know, from BBR-v2 onward, Low Latency, Low Loss, Scalable Throughput (L4S) based Explicit Congestion Notification (ECN) has been added in the BBR-v3 code to take advantage of these markers for better congestion estimation using them. It has a finer-grained response as compared to the traditional ECN. As per the L4S ECN, it reduces the sending rate more intelligently and does more frequent signalling to have a more accurate picture of the network path’s available bandwidth. This helps BBR to make better adjustments in its sending rate and helps in keeping the network queues shallow. It even uses its special codepoint ECT(1) to distinguish L4S traffic from Classic ECN traffic, allowing routers to treat them differently. Unfortunately, L4S is not yet available in the mainline Linux kernel, so we will be reverting to classic ECN. For classic ECN to work properly, it should be enabled on every layer 3 switch or router between the sender and receiver. This is not often the case, and ECN gets a break, and its advantages are not seen. So, for this testing with ECN, we used a simple transparent Fast Ethernet (FE) switch to connect our Linux client to the Linux server running Netserver at port 50,000. ECN was enabled on both the sender and receiver connected via a Fast Ethernet switch. Packet loss was introduced using Linux network emulator (netem) as a Linux queuing discipline (qdisc) within the traffic control (tc) subsystem. Table 6 below shows the throughput comparison between BBR-v3, BBR-n+, and Cubic with increasing packet loss rate (1–5%) without ECN enabled. We see from the bar chart and the corresponding values in the Table 6 that from 1–2% packet loss, the BBR behaved comparatively well in the sense that reasonable throughputs were achieved, but after 2%, there was a marked decrease in the throughput. Results in Table 7 show that when we enabled ECN on both the sender and receiver and performed the same tests by sending a single TCP stream in upload. We see from Table 7 that ECN did improve the throughputs for packet losses up to 2%, but after that, even with ECN enabled, the throughputs have gone further down. BBR’s fallback is to become more conservative to maintain stability, leading to lower throughput. It’s a “better safe than sorry” approach in the face of ambiguity.

**Table 6:** ECN not enabled on 100 Mbps Ethernet.

Packet Loss	Average Throughput (Mbps)		
% (without ECN)	Cubic	BBR-v3	BBR-n+
1%	66.48	68.73	66.28
2%	32.18	27.65	26.57
3%	17.61	19.39	15.83
4%	10.96	15.22	12.41
5%	8.13	6.06	6.25

**Table 7:** ECN enabled on 100 Mbps Ethernet.

Packet Loss	Average Throughput (Mbps)		
% (with ECN)	Cubic	BBR-v3	BBR-n+
1%	71.6	73.06	70.68
2%	34.54	36.02	30.75
3%	16.22	18.86	15.83
4%	10.19	12.93	8
5%	7.97	6.06	6.25

**Fig 24:**
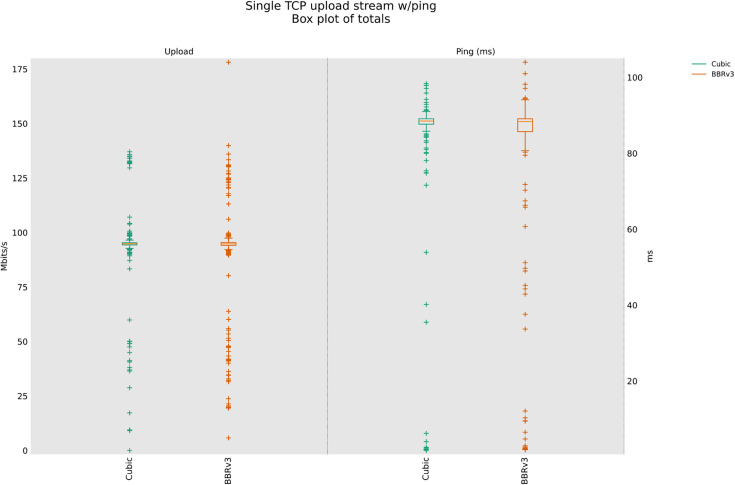
ECN enabled end-to-end, giving full fast Ethernet throughput at 0.5 packet loss.

**Fig 25:**
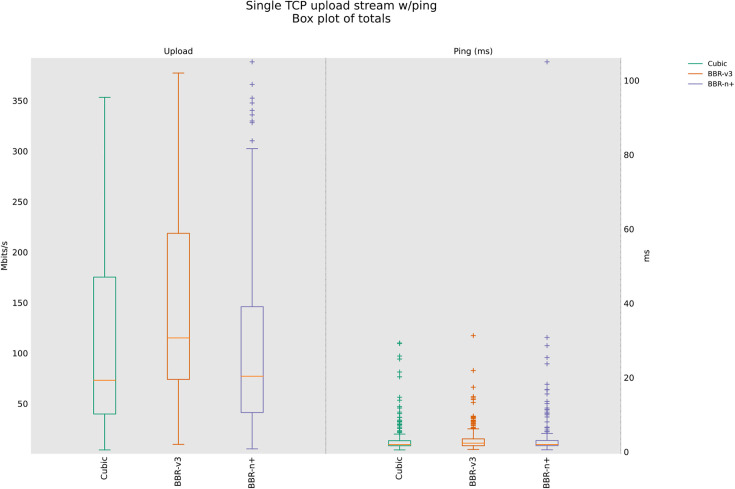
ECN is not enabled end-to-end, and the maximum throughput is not achieved at 0.5 packet loss.

The results of Fig 24 show that with ECN-enabled Fast-Ethernet topology (end-to-end ECN presence), the packet loss is well below 1% i.e, at 0.5% packet loss, the full bandwidth of the Fast Ethernet was achieved. Whereas in the case when ECN is not end-to-end enabled, and there is a layer 3 switch which is not ECN aware, the throughputs are severely hampered, Fig 25. This is a wired Gigabit connected case which gives a maximum of 300 Mbps with no packet loss as shown in Fig 9.

### 4.7 Queue backlog test for CAKE and FQ_Codel (Wireless)

**Fig 26:**
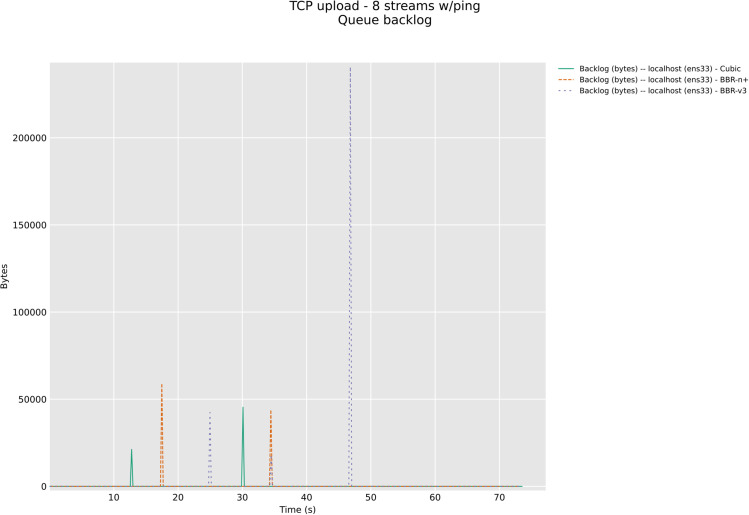
Backlog Bytes with CAKE AQM with 8 streams.

**Fig 27:**
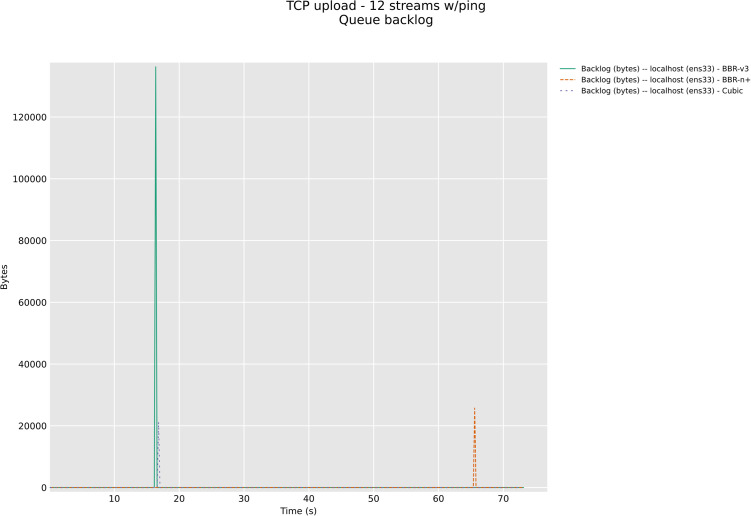
Backlog Bytes with CAKE AQM with 12 streams.

**Fig 28:**
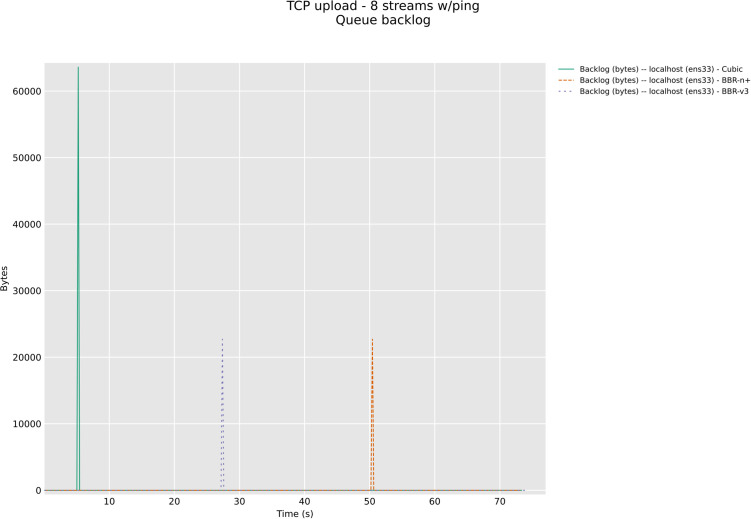
Backlog Bytes with FQ AQM with 8 streams.

In this section, we have evaluated the three CCAs in TCP upload test with 8/12 streams in wireless upload scenario classes with modern AQMs, CAKE, and FQ_CoDel. The Figs 26 and 27 below show backlog bytes for the CAKE AQM under test with 8 and 12 streams in upload. There was no backlog seen with FQ_Codel AQM. To our surprise, the default AQM for BBR-v3, Flow Queuing (FQ), did show backlog bytes for all three CCAs when 8 streams TCP upload test was performed, Fig 28.

**Fig 29:**
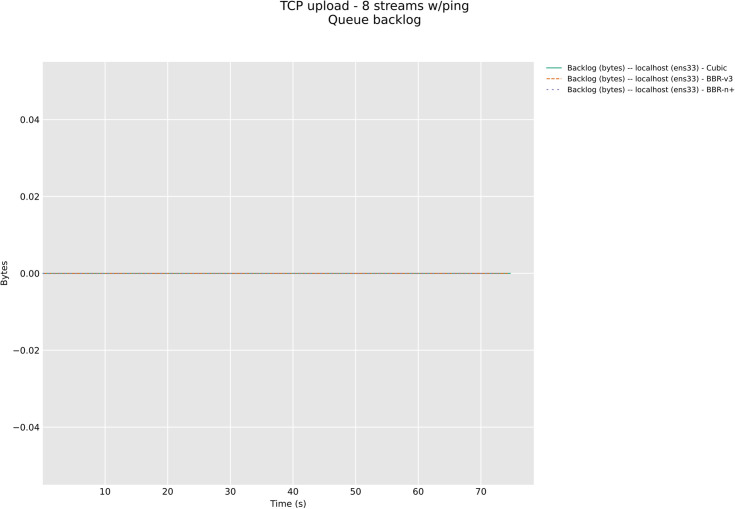
No Backlog with FQ_CoDel AQM with 8 streams.

The significance of queue backlog tests performed in this section with FQ, CAKE, and FQ_CoDel AQMs is that it confirms that FQ_CoDel is indeed the AQM suited for BBR-v3 and BBR-n+, as no backlog bytes were observed with it as the default AQM for the 8 streams upload test, Fig 29. Fair Queuing (FQ), which was good to be used with BBR-v2 here, in the case of BBR-v3 and BBR-n+ gave 5 KB backlog bytes, and 13.3 KB for Cubic. CAKE, which is also a very good AQM for edge devices, especially wireless, gave 69 KB of backlog bytes for BBR-v3 and 21.61 KB for BBR-n+. The loss-based algorithm Cubic here performed well with CAKE and gave only 13.68 KB of backlog bytes. For the sake of brevity, 12 stream tests for FQ_CoDel and others are available in our online repository at GitHub.

With FQ_CoDel, per‑flow scheduling and CoDel’s dropping rule prevent deep queues, reduce ACK distortion, and equalize opportunities for ProbeBW across flows; accordingly, we observe no backlog and improved latency. Our tests further confirm why FQ_CoDel is now the default qdisc in Linux mainline kernel 6.x instead of FQ. These tests will surely motivate the other BBR researchers to try to use FQ_CoDel as the default AQM with BBR and explore it further.

### 4.8 Web (HTTP Delay) test

Our goal was to quantify the delay users experience (HTTP latency) when accessing websites through a network bottleneck, deploying FQ_CoDel and CAKE AQM schemes. This delay encompasses the entire web fetch process: from DNS lookup and retrieving the main page to concurrently downloading all webpage elements via multiple TCP connections. To achieve this, we utilized the client for URLs (cURL) library [49] to simulate client requests. We selected two diverse websites for testing: Google (www.google.com), representing a smaller site with 5 MB across 19 requests, and Huffpost (www.huffpost.com), a larger site with 11 MB over 39 requests. This allowed us to assess how different AQM configurations, particularly our BBR-n+, impact web fetch performance for varying data sizes.

We meticulously fetched the object lists for both sites, repeatedly feeding these URLs to our cURL-based HTTP getter script [50] through the Flent command line. The primary metric we recorded was HTTP delay, measured in milliseconds. To further understand real-world conditions, we also ran these tests with background RRUL traffic and TCP upload traffic. This helped us determine how effectively HTTP requests are delivered under various network loads and, consequently, the impact on the user’s web browsing experience.

**Fig 30:**
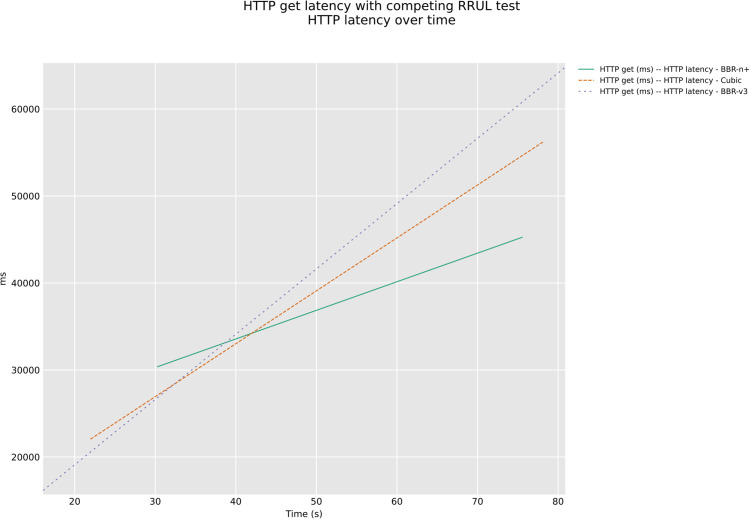
HTTP latency test for Google URLs.

**Fig 31:**
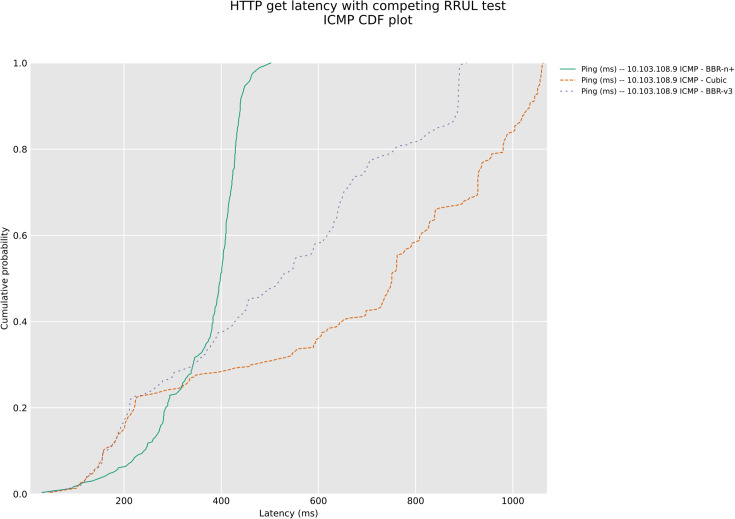
ICMP CDF plot for HTTP latency test for Google URLs.

In a competing HTTP-under-RRUL scenario class with AQM presence, Figs 30 and 31, BBR-n+ demonstrated superior stability in HTTP GET latency for Google URLs with CAKE AQM, exhibiting the slowest rate of latency increase over time. Conversely, BBR-v3, while achieving the lowest initial latency, showed the most aggressive and rapid increase in latency, indicating poor performance in a shared, congested network. Cubic fell between the two, displaying a notable increase in latency but less severe than BBR-v3. These results suggest that BBR-n+ is better suited for maintaining consistent low latency in competitive scenarios, whereas BBR-v3’s aggressiveness can lead to significant latency degradation.

The analysis of both plots of Figs 30 and 31 reveals a clear and consistent performance hierarchy across both HTTP and ICMP latency metrics under the competing RRUL test. BBR-n+ consistently emerges as the superior congestion control algorithm, delivering the lowest latency for both HTTP requests and ICMP packets. BBR-v3 and Cubic show varying performance depending on the metric, but generally, BBR-v3 performs better than Cubic in the ICMP test, while Cubic seems to perform better than BBR-v3 in the HTTP test. This combined analysis highlights the overall effectiveness of BBR-n+ in maintaining low latency and stability, particularly in the presence of competing network traffic.

**Fig 32:**
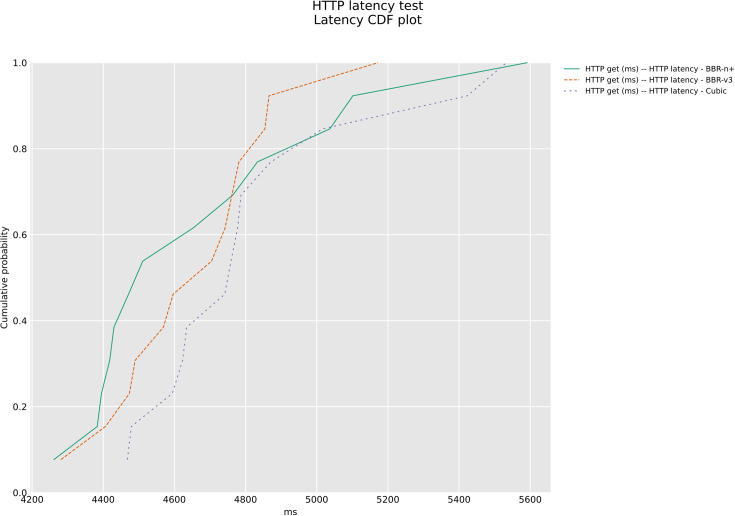
Latency CDF plot for Huffpost URLs with FQ_Codel.

The Cumulative Distribution Function (CDF) plot of Fig 32 compares the HTTP latency performance of BBR-n+, BBR-v3, and Cubic congestion control algorithms. The plot demonstrates that BBR-n+ delivers the lowest latency, with its curve positioned to the left of the others, indicating a higher percentage of requests are completed quickly. BBR-v3 shows intermediate performance, outperforming Cubic but not matching BBR-n+’s efficiency. Cubic consistently exhibits the highest latency, with its curve shifted furthest to the right, signifying the slowest response times and a more significant tail latency. This analysis establishes BBR-n+ as the most effective algorithm for minimizing HTTP latency under the tested conditions.

### 4.9 BBR-n+ and Cubic Fairness test

To evaluate fairness between BBR-n+ and Cubic streams, we used Jain’s fairness Index. Jain’s Fairness Index (JFi) is a widely used metric to assess the fairness of resource distribution among multiple data flows, assuming each flow has identical data rate requirements. The instantaneous fairness index JFi is defined using the data rates R_i_ as follows:


$$JFi\left(R_{\text{1}},R_{\text{2},}\ldots \ldots \ldots R_{n}\right)=\frac{{\left(\sum_{i=\text{1}}^{n}R_{i}\right)}^{\text{2}}}{n.\sum_{i=\text{1}}^{n}\overset{\text{2}}{\underset{i}{R}}}$$

**Fig 33:**
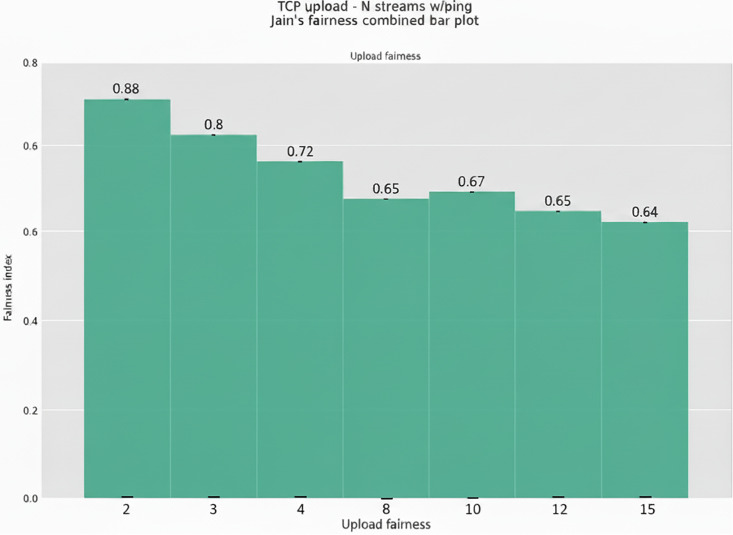
Jain’s fairness Index for various BBR-n+, Cubic streams.

Where n is the number of active flows, R_i_ is the instantaneous date rate of flow i and JFi is a real number in the interval [1/n, 1] with a best-case value of 1 if the data rate is qual for all flows, i.e., the available bandwidth has been fairly shared, and a minimum case of 1/n, if only one aggressive flow is monopolizing the available bandwidth. Fig 33 shows the combined Jain’s fairness Index plot for BBR-n+ and Cubic streams (2,3,4,8,10,12,15) each in upload directions together. The JFi index was initially high when only 2 and 3 streams each of BBR-n+ and Cubic were sent using the TCP upload test. It was 0.88 and 0.8, but when the streams increased to 4,8,10,12, and 15, we saw the JFi started to drop and was only 0.64 for 15 streams of BBR-n+ and 15 streams of Cubic upload test. We see that, like BBR-v3, BBR-n+ still has fairness issues with Cubic when the number of flows is increased from 8 or more.

## 5 Conclusion

This paper evaluated the popular loss-based and model-based algorithms, Cubic, BBR-v3, and BBR-n+ under various test conditions in both wired and wireless scenarios. It is unique in the sense that, alongside evaluations of the three CCAs, it also provides a novel BBR based on a smart exit algorithm. BBR-n+ uses its smart exit algorithm and the algorithms for pacing gain and quantum selection to provide better performance than model-based BBR-v3 and loss-based Cubic on both wired and wireless testbeds Table 2. In the wired testbed case BBR-n+ emerged better with throughputs the same as BBR-v3, but with the lowest and most stable ping latency. Cubic exhibited the highest latency due to the bufferbloat phenomenon in this case Fig 10. In TCP upload test using wireless testbed, BBR-n+ gave superior performance over BBR-v3 but lagged Cubic in the 8/12 streams test. The ICMP ping latency of BBR-n+ was lowest in this case as well Fig 14. In the strenuous RRUL test, BBR-n+ provided better throughput in download and the lowest latency Fig 15. In the wireless scenario for the upload scenario Fig 17, BBR-n+ performed better than the rest of the CCAs. It is due to the smart exit algorithm working in the startup phase, optimized pacing gains, and quantum selections as discussed earlier. BBR-n+ performed better than BBR-v3 in packet loss scenarios and with classic ECN enabled end-to-end Tables 5 and 6. In the queue backlog tests, mainly with modern AQMs CAKE and FQ_CoDel, BBR-n+ gave the least queue backlog bytes as compared to BBR-v3. HTTP delay test for measuring web fetch latency, BBR-n+ gave the least latency Fig 30. BBR-n+ does exhibit fairness issues when concurrent streams are eight or more Fig 33, and we plan to work on this issue in more detail in our future research.

BBR-n+ emerges as the most robust performer in the receiver window limitation case. In wired and wireless cases, it consistently achieves competitive, and often superior, throughput while maintaining significantly lower and more stable ping latencies compared to Cubic and BBR-v3. Cubic shows a strong propensity for bufferbloat and highly variable performance under lossy and multi-stream conditions. BBR-v3 also struggles with consistent performance and latency in these lossy environments, often showing wide distributions and outliers. The ability of BBR-n+ to better manage queuing and react to loss without excessive buffering makes it a more suitable choice for wired and wireless networks experiencing moderate packet loss, especially when real-time application performance is critical.
